# Factors affecting exhaled nitric oxide measurements: the effect of sex

**DOI:** 10.1186/1465-9921-8-82

**Published:** 2007-11-15

**Authors:** D Robin Taylor, Piush Mandhane, Justina M Greene, Robert J Hancox, Sue Filsell, Christene R McLachlan, Avis J Williamson, Jan O Cowan, Andrew D Smith, Malcolm R Sears

**Affiliations:** 1 Department of Respiratory Medicine, Dunedin School of Medicine, University of Otago, Dunedin, New Zealand; 2 Firestone Institute for Respiratory Health, Department of Medicine, McMaster University, Hamilton, Ontario, Canada; 3 Dunedin Multidisciplinary Health and Development Research Unit, Dunedin School of Medicine, University of Otago, Dunedin, New Zealand

## Abstract

**Background:**

Exhaled nitric oxide (F_E_NO) measurements are used as a surrogate marker for eosinophilic airway inflammation. However, many constitutional and environmental factors affect F_E_NO, making it difficult to devise reference values. Our aim was to evaluate the relative importance of factors affecting F_E_NO in a well characterised adult population.

**Methods:**

Data were obtained from 895 members of the Dunedin Multidisciplinary Health and Development Study at age 32. The effects of sex, height, weight, lung function indices, smoking, atopy, asthma and rhinitis on F_E_NO were explored by unadjusted and adjusted linear regression analyses.

**Results:**

The effect of sex on F_E_NO was both statistically and clinically significant, with F_E_NO levels approximately 25% less in females. Overall, current smoking reduced F_E_NO up to 50%, but this effect occurred predominantly in those who smoked on the day of the F_E_NO measurement. Atopy increased F_E_NO by 60%. The sex-related differences in F_E_NO remained significant (p < 0.001) after controlling for all other significant factors affecting F_E_NO.

**Conclusion:**

Even after adjustment, F_E_NO values are significantly different in males and females. The derivation of reference values and the interpretation of F_E_NO in the clinical setting should be stratified by sex. Other common factors such as current smoking and atopy also require to be taken into account.

## Background

Measurement of exhaled nitric oxide (F_E_NO) is increasingly recognised as an important addition to pulmonary function testing in clinical practice [1]. F_E_NO may be used as a surrogate marker for airway eosinophilia [2], and as an alternative to other more invasive or time-consuming assessments of airway pathology such as induced sputum, [2,3] bronchial lavage fluid, [4] or mucosal biopsy. [5-7] Against this background, F_E_NO measurements are increasingly being used to clarify the aetiology of non-specific respiratory symptoms as well as monitor levels of inflammation in conditions characterised by airway eosinophilia [8].

There are a number of demographic and biological factors which cause variation in F_E_NO levels. The commonest are cigarette smoking [9,10] and atopy [11-13] with or without allergic rhinitis. [13,14] Others include age [15,16], and IgE levels [17]. However, conflicting results concerning the importance of these factors has precluded a clear definition of so-called "normal" values. Buchvald et al. have reported reference values in a large population of children, but important biological confounders were evaluated only by questionnaire. [15] The same issues were addressed more recently in adults by Olin et al. [18] and Travers et al. [19], and Travers et al. provided reference ranges which sought to take account of commonly encountered variables which affect F_E_NO. However, there are some significant inconsistencies between these reports, not least in respect of the effects of sex on F_E_NO. Clearly further data are needed so that routine F_E_NO measurements can be interpreted appropriately. In the present study, comprising a well characterised cohort of nearly 1000 32-year old individuals born in Dunedin, New Zealand, we obtained detailed clinical and laboratory information regarding factors affecting F_E_NO, and their potential relevance to reference ranges for F_E_NO was evaluated.

## Methods

The Dunedin Multidisciplinary Health and Development Study is a cohort study of 1037 children (52% male) born between April 1972 and March 1973. [20,21] Follow-up assessments have been conducted at ages 3, 5, 7, 9, 11, 13, 15, 18, 21, 26, and at 32 years, at which time 972 (96%) of 1015 living Study members participated.

At age 32, Study members were questioned about current and previous asthma, as well as symptoms of wheezing, cough, episodic shortness of breath, hay fever and rhinitis. Current asthma was defined as reported diagnosed asthma with symptoms in the last 12 months. Current wheezing was recorded as any wheeze in the last 12 months but excluding subjects with only one or two episodes each lasting less than 1 hour. Asthma treatment was any inhaled bronchodilator, corticosteroid or cromoglycate medication. Current smoking was defined as smoking tobacco cigarettes daily for at least one month during the previous 12 months, or smoking cannabis 6 or more times during the previous 12 months. Current smokers were further subdivided into two groups: those who did and did not smoke on the study day. Ex-smokers were defined as having discontinued for at least 12 months.

Height and weight in light clothing without shoes were measured to calculate body mass index (BMI) in kg/m^2^. F_E_NO was then measured on-line using a Logan LR 2000 series chemiluminescence analyser (Logan Research Ltd., Rochester, England) in accordance with ATS/ERS guidelines at a flow rate of 50 mL/sec. [22] Exhaled nitric oxide in parts per billion (ppb) was recorded continuously throughout expiration. Individual results were read at the first nitric oxide plateau and the mean of two acceptable tests was recorded. A third was obtained only where one or both of the first two were considered to be technically unsatisfactory. The NO recording was determined for each test by two observers on a separate occasion. The first 44 Study members were tested using a flow rate of 250 mL/second and results were adjusted to 50 mL/second using a previously validated formula [23].

F_E_NO measurements were obtained immediately prior to carrying out spirometry. Skin prick testing included house dust mite (*D. pteronyssinus*), grass, cat, dog, horse, cockroach, wool, *Aspergillus fumigatus, alternaria, penicillium*, and *cladosporium*. A weal diameter 3 mm or greater than the saline control was considered positive. Atopy was defined as a positive response to one or more allergens. A blood sample was obtained for eosinophil count and total serum immunoglobulin E (IgE).

### Statistical analysis

Study members who were pregnant at the time of assessment (n = 31) were excluded from all analyses. F_E_NO measurements were not normally distributed, and were log transformed prior to analysis. Both univariate and multivariate linear regression analyses were performed to identify those factors which significantly affected F_E_NO levels and to derive regression equations, with stratification for those factors which were shown to significantly affect exhaled nitric oxide levels. The selection of appropriate linear regression models was based on maximum R-square and an examination of the residuals, to ensure an adequate model fit. Significant interaction terms (p < 0.05) were retained in the model. Results are presented as anti-log values with 95% confidence intervals following back-transformation.

### Ethics

The Otago Ethics Committee approved the study and written informed consent was obtained.

## Results

Eight hundred and ninety-five Study members completed the respiratory procedures in the Study. Of these, 471 (52.6%) were male, 486 (54.8%) were atopic, 349 (39.0%) had rhinitis/hay fever, 253 (28.3%) had current wheeze, 156 (17.4%) had current asthma, and 54 (6.6%) were using inhaled corticosteroid treatment. Three hundred and ninety five were current smokers (44.1%), of whom 235 (59.5%) smoked on the study assessment day prior to testing, and 107 were ex-smokers (12.0%). Two hundred and fifteen were cannabis smokers (24%), of whom 78 (8.7%) smoked cannabis alone.

The F_E_NO values obtained from the Study population are shown in Table 1. Data relating to smoking status are shown in Table 2. F_E_NO was on average 25% higher in males than females (males: 15.3 ppb [95% C.I.:14.3–16.3] versus females: 11.6 ppb [95% C.I.: 11.0–12.4]; p < 0.0001; Table 1). Unadjusted analyses revealed that, for all Study members, there were also significant differences in F_E_NO in relation to height, FEV_1_, FEV_1 _% predicted and FVC (Table 3). However, none of these factors remained significant after stratifying by sex. In contrast, current smoking (on the day of testing), atopy, log IgE, history of rhinitis, and current asthma and the use of inhaled corticosteroids remained significant in both males and females.

**Table 1:** Mean values (with 95% confidence intervals) for F_E_NO stratified by sex, smoking, atopy, rhinitis current wheeze and asthma. * not all Study members underwent skin testing

**All**	All subjects, n = 895 13.4 (12.9, 14.1)											
**Gender**	Male, n = 471 15.3 (14.3,16.3)						Female, n = 424 11.6 (11.0, 12.4)					

**Smoking**	Current smoker (smoked on the day of testing)		Current smoker (not smoked on the day of testing)		Ex-smoker or non-smoker		Current smoker (smoked on the day of testing)		Current smoker (not smoked on the day of testing)		Ex-smoker or non smoker	

	n = 133 8.8 (7.9, 9.7)		n = 102 16.6 (14.7, 18.9)		n = 236 20.2 (18.6, 21.9)		n = 102 7.4 (6.7, 8.2)		n = 58 12.3 (10.2, 14.7)		n = 264 13.7 (12.8, 14.7)	

**Atopy**	Yes	No	Yes	No	Yes	No	Yes	No	Yes	No	Yes	No

	n = 68 9.9 (8.6,11.5)	n = 62 7.6 (6.7, 8.7)	n = 61 19.8 (16.6, 23.6)	n = 41 12.9 (11.1, 14.9)	n = 141 24.8 (22.2, 27.7)	n = 92 14.9 (13.6, 16.3	n = 32 9.5 (7.8, 11.5)	n = 69 6.7 (5.9, 7.5)	n = 35 14.0 (10.6, 18.5)	n = 23 10.0 (8.2, 12.2)	n = 149 15.9 (14.4, 17.6)	n 114 11.2 (10.3, 12.2)

**Rhinitis**	Yes	No	Yes	No	Yes	No	Yes	No	Yes	No	Yes	No

	n = 50 9.2 (8.0, 10.7)	n = 83 8.5 (7.4, 9.7)	n = 30 21.0 (16.0, 27.5)	n = 72 15.1 (13.2, 17.4)	n = 97 24.0 (21.2, 27.2)	n = 139 17.9 (16.1, 19.9)	n = 33 9.0 (7.5,10.9)	n = 69 6.8 (6.0, 7.7)	n = 23 13.4 (10.1, 17.8)	n = 35 11.6 (9.0, 14.9)	n = 116 16.1 (14.3, 18.0)	n = 148 12.1 (11.1, 13.1)

**Current wheeze**	Yes	No	Yes	No	Yes	No	Yes	No	Yes	No	Yes	No

**All**	n = 49 8.4 (6.9, 10.2)	n = 84 9.0 (8.0, 10.0)	n = 35 17.7 (13.4, 23.3)	n = 67 16.1 (14.1, 18.5)	n = 53 28.4 (23.0, 34.9)	n = 183 18.3 (16.9, 19.9)	n = 41 7.6 (6.4, 9.1)	n = 61 7.3 (6.4, 8.4)	n = 18 15.2 (9.4, 24.3)	n = 40 11.1 (9.3, 13.3)	n = 57 16.8 (14.2, 19.8)	n = 207 12.9 (12.0, 13.9)

**Taking ICS**	n = 8 8.0 (4.1, 15.6)	Nil	n = 5 24.0 (12.7, 45.5)	n = 1 72.9	n = 17 27.5 (19.6, 38.7)	n = 2 28.5 (NA)	n = 3 8.8 (2.0, 40.0)	n = 4 9.4 (1.9, 45.2)	n = 3 32.4 (9.3, 112.0)	Nil	n = 13 17.3 (13.1, 22.8)	n = 3 17.8 (3.3, 96.9)

**Not taking ICS**	n = 41 8.5 (6.9, 10.4)	n = 84 9.0 (8.0, 10.0)	n = 30 12.3 (16.8, 22.9)	n = 66 15.8 (13.8, 17.9)	n = 36 28.8 (21.9, 37.8)	n = 181 18.2 (16.8, 19.8)	n = 38 7.5 (6.2, 9.1)	n = 57 7.2 (6.4, 8.2)	n = 15 13.0 (7.7, 22.1)	n = 40 11.1 (9.3, 13.1)	n = 44 16.7 (13.6, 20.5)	n = 204 12.9 (11.9, 13.9)

**Current asthma**	Yes	No	Yes	No	Yes	No	Yes	No	Yes	No	Yes	No

**All**	n = 21 9.8 (6.9, 14.0)	n = 112 8.6 (7.7, 9.5)	n = 27 21.7 (16.5, 28.5)	n = 75 15.1 (13.2, 17.4)	n = 39 29.7 (23.7, 37.2)	n = 197 18.7 (17.2, 20.4)	n = 18 9.5 (6.5, 13.9)	n = 84 7.0 (6.4,7.8)	n = 10 28.0 (14.7, 53.3)	n = 48 10.3 (8.9, 12.0)	n = 41 15.7 (12.7, 19.5)	n = 223 13.3 (12.4, 14.3)

**Taking ICS**	n = 8 8.0 (4.1, 15.6)	Nil	n = 6 28.9 (14.7, 57.0)	Nil	n = 18 26.5 (19.1, 36.8)	n = 1 58.3	n = 5 11.0 (3.8, 32.2)	n = 2 5.7 (0.5, 72.1)	n = 3 32.4 (9.3, 112.0)	Nil	n = 14 18.3 (13.8, 24.3)	n = 2 12.0 (3.1, 47.4)

**Not taking ICS**	n = 13 11.2 (7.1, 17.8)	n = 112 8.6 (7.7, 9.5)	n = 21 20.0 (14.6, 27.4)	n = 75 15.1 (13.2, 17.4)	n = 21 32.7 (23.5, 45.6)	n = 196 18.6 (17.1, 20.2)	n = 13 9.0 (5.8, 14.2)	n = 82 7.1 (6.4, 7.8)	n = 7 26.4 (9.9, 69.9)	n = 48 10.3 (8.9, 12.0)	n = 27 14.6 (10.8, 19.5)	n = 221 13.3 (12.4, 14.4)

**Table 2:** Mean values (with 95% confidence intervals) for F_E_NO, stratified by smoking status and sex

***F_E_NO (ppb) Mean 95% C.I***.	**All subjects**	**Males**	**Females**
**All Study members**	n = 895 13.4 (12.8, 14.1)	n = 471 15.3 (14.3, 16.3)	n = 424 11.6 (11.0, 12.4)
**All current smokers (within last 12 months)**	n = 395 10.4 (9.7, 11.1)	n = 235 11.6 (10.6, 12.6)	n = 160 8.9 (8.1, 9.8)
**Current smokers who smoked on the day of F**_E_**NO testing**	n = 235 8.2 (7.6, 8.8)	n = 133 8.8 (7.9, 9.7)	n = 102 7.4 (6.7, 8.2)
**Current smokers who did not smoke on day of F**_E_**NO testing**	n = 160 14.9 (13.4, 16.6)	n = 102 16.6 (14.7, 18.9)	n = 58 12.3 (10.2, 14.7)
**Ex-smokers (greater than 12 months)**	n = 107 16.0 (14.3, 17.8)	n = 36 21.1 (17.6, 25.3)	n = 71 13.8 (12.2, 15.7)
**Never smokers**	n = 393 16.6 (15.6, 17.7)	n = 200 20.0 (18.3, 22.0)	n = 193 13.6 (12.5, 14.8)

**Table 3:** Factors affecting F_E_NO by linear regression analysis, without controlling for any other factors Magnitude of effect = change compared to reference group (females, non-smokers, non-atopics, non-rhinitics, non-wheezers, or non-asthmatics)

	**All**		**Males**		**Females**	
**Factor**	Magnitude of effect*	**Significance**	Magnitude of effect*	**Significance**	Magnitude of effect*	**Significance**

**Female Gender**	0.7605	<0.0001	---		---	
**BMI**	1.0015	0.7571	1.0045	0.5600	0.9966	0.5503
**Height**	1.0013	<0.0001	1.0003	0.5859	1.0007	0.0969
**FEV**_1_	1.1315	<0.0001	0.9956	0.9328	1.0229	0.7230
**FEV**_1_**% predicted**	0.9957	0.0166	0.9988	0.6422	0.9981	0.4432
**FVC**	1.1058	<0.0001	0.9832	0.6963	1.0443	0.4044
**Current smoker (smoked on day of testing)**	0.49600	<0.0001	0.43350	<0.0001	0.54337	<0.0001
**Current smoker (within last 12 months, not smoked on day of testing)**	0.90579	0.0825	0.82377	0.0091	0.89582	0.1945
**Atopy (≥3 mm)**	1.6028	<0.0001	1.60363	<0.0001	1.5472	<0.0001
**Log IgE**	1.3897	<0.0001	1.3971	<0.0001	1.3235	<0.0001
**Current rhinitis**	1.3126	<0.0001	1.2868	0.0002	1.3670	<0.0001
**Current wheeze**	1.1009	0.0617	1.0906	0.2349	1.1004	0.1658
**Current asthma**	1.4132	<0.0001	1.4434	<0.0001	1.3495	0.0003
**Using ICS**	1.3904	0.0004	1.3780	0.0134	1.3755	0.0130

In the adjusted regression analyses, significant predictors of F_E_NO were sex, body mass index (BMI), current smoking (on the day of testing), atopy, current asthma, and the interaction between sex and smoking. Current wheeze was not a significant factor. This was perhaps because of the significant confounding relationship between current smoking (resulting in reduced F_E_NO) and wheeze (p = 0003; model 4; see Additional File 1). Details of all the models examined are provided in Additional File 1.

After controlling for all of the significant factors affecting F_E_NO, the sex-related differences in F_E_NO remained significant (p < 0.001). The factors which significantly affected F_E_NO were different for males and females. For males, current smoking (all), current asthma, and atopy (any positive SPT ≥ 3 mm over the negative control) were significant independent predictors of F_E_NO. For females, while current smoking (all), current asthma, and atopy (any positive SPT ≥ 3 mm over the negative control) were significant independent predictors of F_E_NO, those females who were current smokers and also had asthma had an additional increase in their F_E_NO (F_E_NO increased by 131%; p = 0.001; Table 4).

**Table 4:** Adjusted linear regression models with F_E_NO as the dependent variable

**Sample**	**Variables**	**Anti-log B-coefficient**	**p-value**	**R-square**
*All Study Members*	Intercept	15.9	<0.0001	0.331
	Sex	0.69	<0.0001	
	Current Smoking – smoked on the testing day	0.45	<0.0001	
	Current Smoking – not smoked on the testing day	0.80	0.0014	
	Current Asthma	1.26	<0.0001	
	Atopy	1.41	<0.0001	
	Sex*Current Smoking – smoked on the testing day	1.32	0.0025	
	Sex*Current Smoking – not smoked on the testing day	1.10	0.3970	

*Males*	Intercept	15.60	<0.0001	0.343
	Current Smoking – smoked on the testing day	0.45	<0.0001	
	Current Smoking – not smoked on the testing day	0.81	0.0021	
	Current Asthma	1.25	0.0025	
	Atopy	1.45	<0.0001	

*Females*	Intercept	11.31	<0.0001	0.276
	Current Smoking – smoked on the testing day	0.57	<0.0001	
	Current Smoking – not smoked on the testing day	0.77	0.0022	
	Current Asthma	1.09	0.3460	
	Atopy	1.37	<0.0001	
	Current Asthma*Current Smoking – smoked on the testing day	1.13	0.4722	
	Current Asthma*Current Smoking – not smoked on the testing day	2.31	<0.0001	

Based on these results, the equations for predicting F_E_NO in our study cohort were:

*For males*: log F_E_NO = 1.1932 - 0.3496*current smoking (smoked day of testing) - 0.0940*current smoking (not smoked day of testing) + 0.16511*atopy + 0.0973*asthma

(R^2 ^= 0.3434)

*For females: *log F_E_NO = 1.0533 - 0.2407*current smoking (smoked day of testing) - 0.1160*current smoking (not smoked on day of testing) + 0.0388*asthma + 0.1355*atopy + 0.0531*current smoking (smoked day of testing)*asthma + 0.3630*current smoking (not smoked day of testing)*asthma

(R^2 ^= 0.2760)

where, for the terms current smoking, atopy and asthma, yes = 1, and no = 0.

Using these equations, the predicted values and ranges (95% C.I.) for clinically important populations are presented in Table 5. For comparison, the actual values obtained from each subgroup of the study population are also presented.

**Table 5:** Mean values and reference ranges for F_E_NO (with 95% confidence intervals), based on prediction equations for males and females. For comparison, the measured values (with 95% confidence intervals) obtained in the Study members are provided

		**Males**		**Females**	
		
**Population**		**F**_E_**NO (ppb)**	**95% C.I**.	**F**_E_**NO(ppb)**	**95% C.I**.
**Non-smokers, non-atopic, non-asthmatic**	**Predicted**	15.6	14.1, 17.2	11.3	10.3, 12.4
	***Actual***	14.7	13.4, 16.1	11.2	10.3, 12.2
**Non-smokers, atopic, non-asthmatic**	**Predicted**	22.6	18.3, 28.0	15.4	12.6, 18.9
	***Actual***	23.1	20.4, 26.2	15.6	13.9,, 17.4
**Non-smokers, non-atopic, asthmatic**	**Predicted**	19.5	15.3, 24.9	12.4	9.4, 16.3
	***Actual***	22.5	8.0, 63.8	11.5	7.0, 18.9
**Non-smokers, atopic, asthmatic**	**Predicted**	28.3	19.8, 40.5	16.9	11.5, 24.9
	***Actual***	30.4	23.9, 38.7	17.2	13.5, 22.1
**Smokers (not smoked on the day of testing), non-atopic, non-asthmatic**	**Predicted**	12.6	9.9, 18.2	8.7	6.7, 11.3
	***Actual***	13.0	11.0, 15.2	9.6	7.8, 11.7
**Smokers (not smoked on the day of testing), atopic, non-asthmatic**	**Predicted**	18.2	12.8, 25.5	11.8	8.2, 17.2
	***Actual***	17.6	14.1, 22.0	10.9	8.7, 13.8
**Smokers (not smoked on the day of testing), non-atopic, asthmatic**	**Predicted**	15.7	10.7, 22.8	21.8	9.2, 51.8
	***Actual***	12.0	7.5, 19.5	16.0	0.3, 743.5
**Smokers (not smoked on the day of testing), atopic, asthmatic**	**Predicted**	22.8	13.6, 36.9	29.8	11.3, 79.0
	***Actual***	24.0	17.8, 32.4	32.3	14.6, 71.2
**Smokers (smoked on the day of testing), non-atopic, non-asthmatic**	**Predicted**	7.0	5.6, 8.8	6.5	5.1, 8.2
	***Actual***	7.3	6.4, 8.3	6.5	5.8, 7.3
**Smokers (smoked on the day of testing), atopic, non-asthmatic**	**Predicted**	10.1	7.2, 14.0	8.9	6.3, 12.5
	***Actual***	10.2	8.8, 11.8	8.9	7.3, 10.9
**Smokers (smoked on the day of testing), non-atopic, asthmatic**	**Predicted**	8.7	6.0, 12.5	8.0	3.8, 17.0
	***Actual***	8.9	7.3, 10.9	8.1	4.0, 16.4
**Smokers (smoked on the day of testing), atopic, asthmatic**	**Predicted**	12.6	7.6, 20.3	11.0	4.6, 26.0
	***Actual***	9.3	6.2, 13.8	10.9	6.6, 18.0

## Discussion

The results of the present study provide further evidence that sex is a major factor determining exhaled nitric oxide (F_E_NO) measurements. Without adjusting for other factors such as atopy, current smoking, and diagnosed asthma, the mean F_E_NO levels in males were significantly higher than in females (p = 0.0001). However, even after appropriate adjustments, this difference persisted. The magnitude of the difference was approximately 25%. This is clinically as well as statistically significant [24].

A review of the literature provides somewhat conflicting data regarding this issue. It is important to take account of the different methodologies used for F_E_NO measurements when making comparisons *between *studies, particularly with regard to expiratory flow rates. However, *within *studies, significant differences between males and females will still be valid, and the balance of evidence suggests that sex-related differences in F_E_NO are indeed important. In early investigations, both Jilma et al. [25] and Tsang et al. [26] reported sex-related differences in F_E_NO whose magnitude (50% and 53% higher in males compared to females, respectively) was comparable to the present result. More recently, Olivieri et al. have reported higher levels in males, with an upper limit of normal of 28.8 ppb, compared to 21.5 ppb for females [27]. Travers et al. [19] reported that the mean F_E_NO in males was 23% higher than in females (95% C.I. 7–43; p = 0.004, n = 191). In that study, the significance of the difference persisted even after controlling for height. In the study by Berry et al. a similar highly significant difference between males and females was recorded [28]. However, in the largest study to date to focus on factors affecting F_E_NO, comprising 2,200 subjects, Olin et al. has presented contrasting results [18]. Although there was a male-female F_E_NO difference in non-smokers amounting to 19%, this was not statistically significant in a multiple linear regression analysis in which adjustments for all other factors were included [18]. The reasons why the difference failed to reach statistical significance are unclear.

After adjusting for sex, we found that other anthropometric factors such as height and lung function were no longer significant factors affecting F_E_NO. Previously it has been argued that sex-related differences in F_E_NO result from differences in the surface area of airway epithelium, the major source of exhaled NO, and for which height is an important anthropometric correlate. Thus our results are perhaps surprising. However, given that plasma levels of nitrate, a product of NO metabolism, are similarly different between the sexes [25,29], it seems unlikely that NO production in the airways is solely a reflection of differences in airway size, but rather reflects sex-related differences in endogenous NO production. This is consistent with the results of a twin study, which showed that genetic rather than environmental factors are more important in determining F_E_NO [30].

Our findings raise the question as to whether guidelines for the interpretation of F_E_NO should be stratified by sex, and that reference ranges for males and females should be different. In the paper by Olivieri et al. the authors propose that reference ranges should be stratified for sex [27]. Travers et al. [19] advocate reference ranges based on sex, smoking status and atopy, but not age or height. We concur with this view, and the reference ranges contained in Table 5 of the present paper are based on this approach. In the study by Olin et al. [18], similar to conventional pulmonary function tests, both age and height but not sex, were deemed to be significant, although reference values as such were not provided. All studies concur that smoking and atopy are important considerations, and both are included in the reference values given here and by Travers et al. [19].

In fact, interpreting F_E_NO levels in clinical practice is even more complex. Reference values which take into account background characteristics such as sex, atopy and smoking may indeed be useful in guiding the diagnosis of airways-related *symptoms*. In asymptomatic individuals, it is still possible that increased F_E_NO reflects subclinical airway inflammation [7,28], but this interpretation is less likely if appropriate reference values which take factors such as sex into account have been used in the first place. The interpretation of F_E_NO levels in the context of ongoing management of diagnosed asthma is far from clear. Despite optimal anti-inflammatory treatment, F_E_NO levels may remain resolutely high [31], and it is generally agreed that normalizing F_E_NO in relation to reference values for a healthy population is not a desirable therapeutic aim [8]. This point is perhaps reflected in the results obtained in the present study, which showed that in non-smoking, atopic, male asthmatics, who were all clinically stable, the upper limit of the 95% confidence interval was 38.8 ppb, considerably higher than the levels obtained in non-smoking, atopic, male, non-asthmatics (28.2 ppb).

One of the weaknesses of our study is that the F_E_NO measurements were obtained in individuals who were all aged 32 years. Thus it was not possible to explore the influence of age as a factor in the regression analyses or to conclude whether reference values ought to include it as a factor. Previous studies have reported that F_E_NO rises with increasing age in children [15,16,32-34]. In adults, Olin et al. [18] have reported that an effect of age also occurs: F_E_NO was shown to increase over the age range 35 to 65 years, with the magnitude of effect similar to that of atopy. In contrast, in the study by Travers et al. no significant relationship was noted over a similar age range, but numbers were much smaller [19]. In children, it is suggested that the changes with age are attributable to increasing airway NO flux, probably reflecting larger airway surface area with growth [34]. If at all, any increase in F_E_NO with age in adults is likely to be due to non-anthropometric factors, and if the results from Olin et al. are repeatable, this may be an important consideration.

In summary, our data confirm that differences in F_E_NO between males and females are of sufficient magnitude that the interpretation of F_E_NO should be stratified by sex. This approach should be incorporated into clinical practice. Other common and easily identified factors such as current smoking and atopy also require to be taken into account when interpreting F_E_NO values in adults. Contrasting results from a number of studies still leave open the question as to whether age and height ought to be included in future reference equations. These outstanding issues add to the current challenges which still remain in the application and interpretation of F_E_NO levels in clinical practice, and require further study.

## Abbreviations

C.I. confidence interval

F_E_NO fraction of nitric oxide in expired air

FEV_1 _forced expiratory volume in one second

FVC forced vital capacity

NO nitric oxide

mL/sec milliliters per second

ppb parts per billion

SPT skin prick test

## Competing interests

D Robin Taylor has received lecture fees and an unrestricted educational grant valued at $20,000, from Aerocrine, a manufacturer of nitric oxide analysers

All other named authors have no competing interests to declare in relation to the contents of this manuscript.

## Authors' contributions

**D Robin Taylor **devised the study plan and wrote the manuscript

**Piush Mandhane **conducted statistical analysis of the results and contributed to writing the manuscript

**Justina M Greene **conducted statistical analysis of the results

**Robert J. Hancox **directed and supervised the conduct of the study and data collection

**Christene R McLachlan **provided technical assistance in the collection of study data

**Avis J Williamson **provided technical assistance in the collection of study data

**Sue Filsell **provided technical assistance in the collection of study data

**Jan O Cowan **provided technical assistance in the collection of study data

**Andrew D Smith **provided technical assistance in the collection of study data

**Malcolm R Sears **provided general study oversight and contributed to writing the manuscript

All named authors have approved the contents of this manuscript.

## Supplementary Material

Additional File 1 Selection of adjusted linear regression models for all study members, and stratified by males and females Click here for file
