# Supplementary material for: Factors affecting exhaled nitric oxide measurements: the effect of sex
Source: Respir Res. 2007 Nov 15;8(1):82. doi: 10.1186/1465-9921-8-82 (PMC2231356; doi:10.1186/1465-9921-8-82)
Supplement: Additional File 1 — Selection of adjusted linear regression models for all study members, and stratified by males and females [file 1465-9921-8-82-S1.doc]

Selection of adjusted linear regression models for all study members, and stratified by males and females

| Sample | Model | Variables | Β-oefficient | p-value | R-Square |
| --- | --- | --- | --- | --- | --- |
| All Study Members | 1 | Intercept | 1.2305 | <.0001 | 0.3412 |
|  |  | Sex | -0.1700 | <.0001 |  |
|  |  | Current Smoking– smoked day of testing | -0.3547 | <.0001 |  |
|  |  | Current Smoking – not smoked day of testing | -0.1257 | 0.0001 |  |
|  |  | Current Asthma | 0.09185 | 0.0031 |  |
|  |  | Atopy | 0.1479 | <.0001 |  |
|  |  | BMI | -0.0424 | 0.0126 |  |
|  |  | Sex*Current Smoking– smoked day of testing | 0.1265 | 0.0015 |  |
|  |  | Sex*Current Smoking– not smoked day of testing | 0.0470 | 0.3147 |  |
|  |  | Current Asthma* Current Smoking– smoked day of testing | -0.0359 | 0.5017 |  |
|  |  | Current Asthma* Current Smoking– not smoked day of testing | 0.1020 | 0.0672 |  |
|  |  |  |  |  |  |
|  | 2 | Intercept | 1.2284 | <.0001 | 0.3374 |
|  |  | Sex | -0.1698 | <.0001 |  |
|  |  | Current Smoking– smoked day of testing | -0.3601 | <.0001 |  |
|  |  | Current Smoking – not smoked day of testing | -0.1001 | 0.0007 |  |
|  |  | Current Asthma | 0.1057 | <.0001 |  |
|  |  | Atopy | 0.1477 | <.0001 |  |
|  |  | BMI | -0.0425 | 0.0125 |  |
|  |  | Sex*Current Smoking – smoked day of testing | 0.1251 | 0.0018 |  |
|  |  | Sex*Current Smoking– not smoked day of testing | 0.0389 | 0.4045 |  |
|  |  |  |  |  |  |
|  | 3 | Intercept | 1.2278 | <.0001 | 0.3374 |
|  |  | Sex | -0.1687 | <.0001 |  |
|  |  | Current Smoking– smoked day of testing | -0.3599 | <.0001 |  |
|  |  | Current Smoking – not smoked day of testing | -0.1000 | 0.0008 |  |
|  |  | Current Asthma | 0.1058 | <.0001 |  |
|  |  | Atopy | 0.1476 | <.0001 |  |
|  |  | BMI | -0.0396 | 0.4582 |  |
|  |  | BMI*Sex | -0.0020 | 0.9541 |  |
|  |  | Sex*Current Smoking– smoked day of testing | 0.1248 | 0.0020 |  |
|  |  | Sex*Current Smoking– not smoked day of testing | 0.0387 | 0.4081 |  |
|  |  |  |  |  |  |
|  | 4 | Intercept | 1.2001 | <.0001 | 0.3320 |
|  |  | Sex | -0.1637 | <.0001 |  |
|  |  | Current Smoking– smoked day of testing | -0.3506 | <.0001 |  |
|  |  | Current Smoking– not smoked day of testing | -0.0946 | 0.0014 |  |
|  |  | Current Asthma | 0.1020 | <.0001 |  |
|  |  | Atopy | 0.1485 | <.0001 |  |
|  |  | Sex*Current Smoking– smoked day of testing | 0.1209 | 0.0025 |  |
|  |  | Sex*Current Smoking– not smoked day of testing | 0.0394 | 0.3970 |  |
|  |  |  |  |  |  |
|  | 5 | Intercept | 1.1989 | <.0001 | 0.3219 |
|  |  | Sex | -0.1641 | <.0001 |  |
|  |  | Current Smoking– smoked day of testing | -0.3583 | <.0001 |  |
|  |  | Current Smoking– not smoked day of testing | -0.0907 | 0.0023 |  |
|  |  | Current Wheeze | 0.0533 | 0.0058 |  |
|  |  | Atopy | 0.1587 | <.0001 |  |
|  |  | Sex*Current Smoking– smoked day of testing | 0.1235 | 0.0022 |  |
|  |  | Sex*Current Smoking– not smoked day of testing | 0.0321 | 0.4930 |  |
|  |  |  |  |  |  |
|  |  |  |  |  |  |
|  | 6 | Intercept | 1.1858 | <.0001 | 0.3310 |
|  |  | Sex | -0.1633 | <.0001 |  |
|  |  | Current Smoking– smoked day of testing | -0.3124 | <.0001 |  |
|  |  | Current Smoking– not smoked day of testing | -0.0760 | 0.0228 |  |
|  |  | Current Wheeze | 0.1125 | <.0001 |  |
|  |  | Atopy | 0.1579 | <.0001 |  |
|  |  | Current wheeze*Current Smoking– smoked day of testing | 0.1489 | 0.0006 |  |
|  |  | Current wheeze*Current Smoking– not smoked day of testing | 0.0628 | 0.2064 |  |
|  |  | Sex*Current Smoking– smoked day of testing | 0.1265 | 0.0016 |  |
|  |  | Sex*Current Smoking– not smoked day of testing | 0.0312 | 0.5037 |  |
|  |  |  |  |  |  |
| By Sex | Males | Intercept | 1.2090 | <.0001 | 0.3509 |
|  |  | Current Smoking– smoked day of testing | -0.3386 | <.0001 |  |
|  |  | Current Smoking– not smoked day of testing | -0.0922 | 0.0083 |  |
|  |  | Current Asthma | 0.1567 | 0.0092 |  |
|  |  | Atopy | 0.1604 | <.0001 |  |
|  |  | BMI | -0.0369 | 0.2305 |  |
|  |  | Current Asthma * Bmi | -0.0340 | 0.5836 |  |
|  |  | Current Smoking * Current Asthma– smoked day of testing | -0.1181 | 0.1247 |  |
|  |  | Current Smoking * Current Asthma– not smoked day of testing | -0.0393 | 0.5899 |  |
|  |  |  |  |  |  |
|  | Females | Intercept | 1.0646 | <.0001 | 0.2965 |
|  |  | Current Smoking– smoked day of testing | -0.2426 | <.0001 |  |
|  |  | Current Smoking– not smoked day of testing | -0.1214 | 0.0014 |  |
|  |  | Current Asthma | 0.1242 | 0.0175 |  |
|  |  | Atopy | 0.1367 | <.0001 |  |
|  |  | BMI | -0.0244 | 0.3331 |  |
|  |  | Current Asthma * Bmi | -0.1502 | 0.0174 |  |
|  |  | Current Asthma* Current Smoking – smoked day of testing | 0.0353 | 0.6312 |  |
|  |  | Current Asthma *Current Smoking– not smoked day of testing | 0.3756 | <.0001 |  |
|  |  |  |  |  |  |
|  | Males | Intercept | 1.2216 | <.0001 | 0.3473 |
|  |  | Current Smoking– smoked day of testing | -0.3589 | <.0001 |  |
|  |  | Current Smoking– not smoked day of testing | -0.0990 | 0.0013 |  |
|  |  | Current Asthma | 0.0972 | 0.0025 |  |
|  |  | Atopy | 0.1599 | <.0001 |  |
|  |  | BMI | -0.0413 | 0.0911 |  |
|  |  |  |  |  |  |
|  | Females | Intercept | 1.0654 | <.0001 | 0.2589 |
|  |  | Current Smoking– smoked day of testing | -0.2386 | <.0001 |  |
|  |  | Current Smoking– not smoked day of testing | -0.0609 | 0.0839 |  |
|  |  | Current Asthma | 0.1139 | 0.0005 |  |
|  |  | Atopy | 0.1349 | <.0001 |  |
|  |  | BMI | -0.0441 | 0.0626 |  |
|  |  |  |  |  |  |
|  | Males | Intercept | 1.1932 | <.0001 | 0.3434 |
|  |  | Current Smoking– smoked day of testing | -0.3496 | <.0001 |  |
|  |  | Current Smoking– not smoked day of testing | -0.0940 | 0.0021 |  |
|  |  | Current Asthma | 0.0973 | 0.0025 |  |
|  |  | Atopy | 0.1611 | <.0001 |  |
|  |  |  |  |  |  |
|  | Females | Intercept | 1.0434 | <.0001 | 0.2487 |
|  |  | Current Smoking– smoked day of testing | -0.2331 | <.0001 |  |
|  |  | Current Smoking– not smoked day of testing | -0.0547 | 0.1182 |  |
|  |  | Current Asthma | 0.10538 | 0.0013 |  |
|  |  | Atopy | 0.1351 | <.0001 |  |
|  |  |  |  |  |  |
|  | Males | Intercept | 1.1864 | <.0001 | 0.3470 |
|  |  | Current Smoking– smoked day of testing | -0.3303 | <.0001 |  |
|  |  | Current Smoking– not smoked day of testing | -0.0882 | 0.0112 |  |
|  |  | Current Asthma | 0.1376 | 0.0027 |  |
|  |  | Atopy | 0.1612 | <.0001 |  |
|  |  | Current Asthma* Current Smoking – smoked day of testing | -0.1220 | 0.1107 |  |
|  |  | Current Asthma* Current Smoking – not smoked day of testing | -0.0367 | 0.6129 |  |
|  |  |  |  |  |  |
|  | Females | Intercept | 1.0533 | <.0001 | 0.2760 |
|  |  | Current Smoking– smoked day of testing | -0.2407 | <.0001 |  |
|  |  | Current Smoking– not smoked day of testing | -0.1160 | 0.0022 |  |
|  |  | Current Asthma | 0.0388 | 0.3460 |  |
|  |  | Atopy | 0.1355 | <.0001 |  |
|  |  | Current Asthma *Current Smoking – smoked day of testing | 0.0531 | 0.4722 |  |
|  |  | Current Asthma *Current Smoking– not smoked day of testing | 0.3630 | <.0001 |  |
|  |  |  |  |  |  |
|  | Males | Intercept | 1.2022 | <.0001 | 0.3495 |
|  |  | Current Smoking– smoked day of testing | -0.3126 | <.0001 |  |
|  |  | Current Smoking– not smoked day of testing | -0.0620 | 0.0912 |  |
|  |  | Current Wheeze | 0.1259 | 0.0022 |  |
|  |  | Atopy | 0.1681 | <.0001 |  |
|  |  | Bmi32 | -0.0382 | 0.1202 |  |
|  |  | Current Wheeze* Current Smoking- smoked day of testing | -0.1750 | 0.0048 |  |
|  |  | Current Wheeze* Current Smoking– not smoked day of testing | -0.1216 | 0.0680 |  |
|  |  |  |  |  |  |
|  | Females | Intercept | 1.0526 | <.0001 | 0.2590 |
|  |  | Current Smoking– smoked day of testing | -0.2028 | <.0001 |  |
|  |  | Current Smoking– smoking on day of testing | 0.0795 | 0.0596 |  |
|  |  | Current Wheeze | 0.1040 | 0.0047 |  |
|  |  | Bmi32 | -0.0419 | 0.0769 |  |
|  |  | Atop3mm32 | 0.1469 | <.0001 |  |
|  |  | Current wheeze* Current Smoking– smoked day of testing | -0.1224 | 0.0453 |  |
|  |  | Current wheeze* Current Smoking– not smoked day of testing | 0.0321 | 0.6793 |  |
|  |  |  |  |  |  |
|  | Males | Intercept | 1.1756 | <.0001 | 0.3461 |
|  |  | Current Smoking– smoked day of testing | -0.3018 | <.0001 |  |
|  |  | Current Smoking– not smoked day of testing | -0.0566 | 0.1213 |  |
|  |  | Current Wheeze smoked day of testing | 0.1268 | 0.0020 |  |
|  |  | Atopy | 0.1695 | <.0001 |  |
|  |  | Current wheeze* Current Smoking– smoking on day of testing | -0.1809 | 0.0034 |  |
|  |  | Current wheeze* Current Smoking– not smoked day of testing | -0.1241 | 0.0627 |  |
|  |  |  |  |  |  |
|  | Females | Intercept | 1.0327 | <.0001 | 0.2494 |
|  |  | Current Smoking– smoked day of testing | -0.2008 | <.0001 |  |
|  |  | Current Smoking– not smoked day of testing | -0.0733 | 0.0793 |  |
|  |  | Current Wheeze | 0.0967 | 0.0083 |  |
|  |  | Atopy | 0.1460 | <.0001 |  |
|  |  | Current wheeze* Current Smoking– smoked day of testing | -0.1120 | 0.0668 |  |
|  |  | Current wheeze* Current Smoking– not smoked day of testing | 0.0353 | 0.6496 |  |
